# Current Trends and Perspectives on Predictive Models for Mildew Diseases in Vineyards

**DOI:** 10.3390/microorganisms11010073

**Published:** 2022-12-27

**Authors:** Luisa Velasquez-Camacho, Marta Otero, Boris Basile, Josep Pijuan, Giandomenico Corrado

**Affiliations:** 1 Eurecat, Centre Tecnològic de Catalunya, Unit of Applied Artificial Intelligence, 08005 Barcelona, Spain; 2 Department of Crop and Forest Sciences, University of Lleida, 25199 Lleida, Spain; 3 Department of Agricultural Sciences, University of Naples Federico II, 80055 Naples, Italy

**Keywords:** disease modelling, infection forecast, powdery mildew, downy mildew, precision agriculture

## Abstract

Environmental and economic costs demand a rapid transition to more sustainable farming systems, which are still heavily dependent on chemicals for crop protection. Despite their widespread application, powdery mildew (PM) and downy mildew (DM) continue to generate serious economic penalties for grape and wine production. To reduce these losses and minimize environmental impacts, it is important to predict infections with high confidence and accuracy, allowing timely and efficient intervention. This review provides an appraisal of the predictive tools for PM and DM in a vineyard, a specialized farming system characterized by high crop protection cost and increasing adoption of precision agriculture techniques. Different methodological approaches, from traditional mechanistic or statistic models to machine and deep learning, are outlined with their main features, potential, and constraints. Our analysis indicated that strategies are being continuously developed to achieve the required goals of ease of monitoring and timely prediction of diseases. We also discuss that scientific and technological advances (e.g., in weather data, omics, digital solutions, sensing devices, data science) still need to be fully harnessed, not only for modelling plant–pathogen interaction but also to develop novel, integrated, and robust predictive systems and related applied technologies. We conclude by identifying key challenges and perspectives for predictive modelling of phytopathogenic disease in vineyards.

## 1. Introduction

The ambitious need of rapid agro-ecological transition towards more sustainable and resilient agriculture necessitates a strong increase in the use efficiency of plant protection products (PPPs). Tools that can enhance our capacity to control plant infections are also much needed because of the increasing cost of pesticides and the more stringent regulations on use and registration of PPPs. There is a longstanding consensus that the ability to predict the conditions that justify chemical intervention in agriculture is crucial to prevent, restrict, and manage plant pathogens.

A predictive model is any formal representation able to forecast future events or outcomes based on available information (e.g., input data and previous patterns). For plant–pathogen interaction and many other fields, this broad definition embraces several systems, which can be built exploiting an array of approaches and datasets. Nonetheless, while it is generally accepted that predictive systems in agriculture can provide wide-ranging economic, environmental, social, and health benefits, their adoption can be considered, with few exceptions, unsatisfactory [1]. There are several factors that influence the percentage and duration of adoption of predictive systems, but it is undeniable that their implementation in agriculture cannot be disjoined by an improved ability to deliver appropriate management decisions with minimum risk [1]. In the last few decades, development of “omics” technologies has expanded our understanding of the fundamental biological and environmental factors that govern the molecular interaction between plants and microbial pathogens. Similarly, advancements in information technology, computational power, and data science have increased the affordability and reliability of digital solutions. The potential impact of these developments is multifold. They are valuable to study microbial community dynamics, understand plant molecular and cellular responses, identify key risk factors, discover patterns, and formalize algorithms, ultimately increasing the possibility of predicting disease outbreaks timely, robustly, and with a high degree of accuracy and sensitivity.

The aim of this review is to critically examine the trends in predictive models for disease progression in agriculture. We also briefly describe their assumptions and main properties. This paper focuses on grapevine and downy and powdery mildews because this plant species is the most important fruit shrub crop globally [2] and, historically, one of the most employed models to build predictive tools in plant pathology. Moreover, the vineyard is a highly specialized plantation, with a crop protection cost in the EU probably second only to commercial horticulture outdoors. We first introduce the grapevine and the two pathogens to highlight the characteristics that influence their growth and are, therefore, relevant for the model design. We then examine trends from a systematic literature review and outline some of the main models used as predictors of phytopathogenic diseases in vineyards. Finally, we discuss current limitations and highlight that integration of expertise of plant molecular genetics and physiology, botany, physics, data science, and engineering is today a well-timed step to develop novel and more robust predictive systems.

### 1.1. Grapevine and Its Main Phytopathogens

Grape production is an important business worldwide [3]. In the 1982–2022 period, Europe has been the main grape producer (48.4%), followed by Asia (24.9%), America (19%), Africa (5.3%), and Oceania (2.3%). It is notable that, in the last five years, Asia has become the main vine producer, mainly because of the contribution of PR of mainland China (36.8% of world’s total) [2]. Given the historical, economic, and cultural importance of grapevine, it is understandable that, over the years, a plethora of studies have been conducted to anticipate or mitigate crop damage caused by microbial phytopathogens. Different characteristics of the grape farming system include presence of large areas of monoculture, favor emergence of pathogens, and, even in the presence of chemical control methods, they can cause catastrophic results in terms of productivity [4,5].

Downy mildew (DM) and powdery mildew (PM) are, along with gray mold (caused by *Botrytis cinerea*), the most devastating microbial diseases of grapevine [6], reducing yield by an estimated 12% [7]. *Plasmopara viticola* (Berk. & M.A. Curtis) Berl. & De Toni 1888, is the causal agent of DM and arguably the main disease of grapevines [8,9]. This oomycete is probably native to the northeastern USA. DM impacts production through reduction in photosynthetic activity of the affected green tissues, also promoting premature leaf fall and, consequently, reduction in grape quality [8,9,10]. DM control is currently based on containment of oospore germination, the resistance structures of this pathogen between vegetative seasons [11]. *Uncinula necator* (Schwein.) Burrill 1892 is the causal agent of PM, and it also originated in North America. This ascomycete infects green tissues, including berries, causing large yield loss and decreased wine quality. *U. necator* survives in the soil or in the basal part of the bark as cleistothecium (chasmothecium), a completely closed, globose fruit body [12]. The most important and widely cultivated *Vitis vinifera* (L.) varieties are of Eurasian origin and are susceptible because they have only been recently exposed to these pathogens [13,14,15].

From an evolutionary perspective, *P. viticola* and *U. necator* are highly successful despite their relative ability to naturally spread. They have quicky adapted to different host varieties, continents, micro-climates, and farming systems. These two pathogens are typically controlled with recurrent use of fungicide, yet their occurrence has increased in the last decade [15,16]. Agronomic strategies, including use of host plant resistance, have not proved to be effective also because of vegetative propagation of the grapevine varieties and their strong link with specific territories and winemaking, which imposes a constraint on genetic intervention. Regardless of the production system (e.g., conventional or organic), many growers apply fungicides in advance starting from early spring (when the first shoots have not yet appeared) at intervals of 7 to 14 days [9]. The economic problems caused by these phytopathogens, the limitation of use of fungicides when the disease is already in advanced stages of infection, and the environmental and economic costs of their heavy use have prompted development of a series of disease warning systems (WS) or decision support systems (DSS). Although they rely on different mathematical models, they have the common goal to predict disease occurrence and manage application of fungicides [17,18].

### 1.2. Main Stages of the Life Cycle of the Phytopathogens and Their Relationship to Environmental Conditions

#### 1.2.1. *Plasmopara viticola*

**Figure 1:**
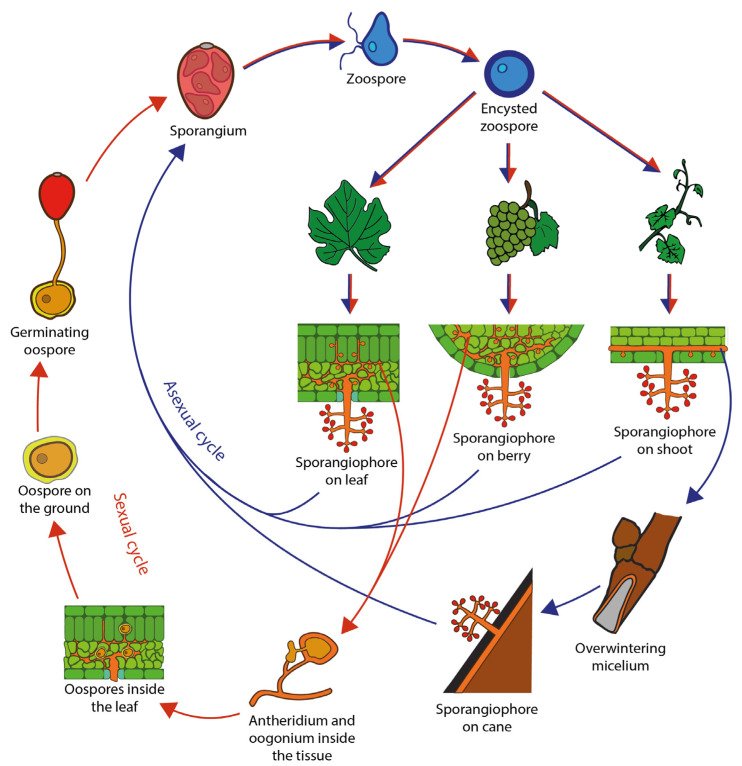
Schematic diagram of the disease cycle of downy mildew caused by *Plasmopara viticola*. Red arrows refer to the sexual cycle. Blue arrows refer to the asexual cycle.

*P. viticola* is nowadays a cosmopolitan plant pathogen. It thrives in humid conditions, and it is an obligate biotrophic endoparasite of the green organs of the vine. During the winter cold, *P. viticola* remains inactive as oospore in fallen infected leaves, using plant debris as shelter (Figure 1). Oospore maturation starts with mild and rainy days in spring, usually with prolonged rainfall (e.g., at least 6 mm), temperature between 6 and 26 °C, and a relative humidity above 90% (Table 1). The germinated oospores form sporangia that, with the help of rain splashes and wind, reach the potential host tissue, normally wet young leaves near the soil. Sporangia releases motile biflagellate zoospores that encyst when in contact with the host plant tissue. The germinated encysted zoospore infects young green tissues through open stomata, and it will produce a mycelium that, by colonizing the intercellular spaces, will cause the appearance of “oil spots”, typically visible on the upper side of the leaves. *P. viticola* will draw nutrients from plant cells through globose haustoria. Asexual reproduction occurs when the mycelium will produce sporangiophores emerging through the stomata of leaves, shoots, or the lenticels of young fruits. Sporangia are released from the tip of the sporangiophores’ branches, and the released zoospores will start a secondary infection cycle. The initial oil spot lesions usually appear at this stage as a brownish area spreading on the infected tissue. The whole cycle (up to production of sporangiophores) typically lasts from less than one week to 18 days [14,17,19,20]. Under favorable conditions (e.g., rain and wind speed stronger than 9 m s^−1^), the infection cycle occurs repeatedly during the growing season [21]. With lowering temperatures, the fungal sexual reproduction inside the infected tissues (mainly leaves) will produce numerous oospores, the thick-walled resting structures that can last years in leaf litter on the vineyard floor. In some instances, the pathogen overwinters as mycelium in infected thin branches (twigs).

#### 1.2.2. *Uncinula necator*

**Figure 2:**
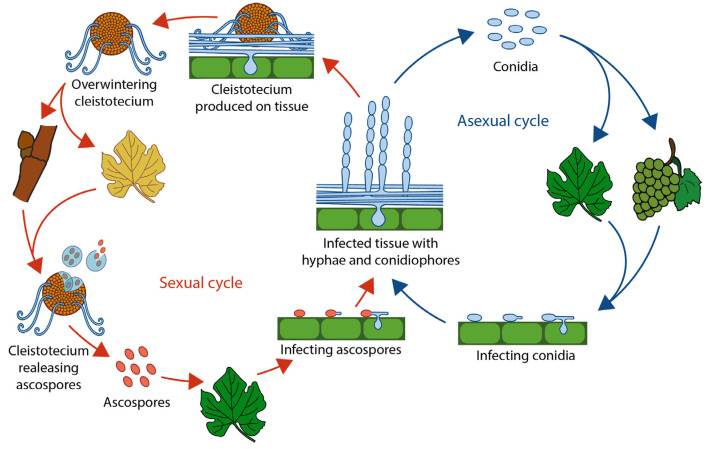
Schematic diagram of the disease cycle of powdery mildew caused by *Uncinula necator*. Red arrows refer to the sexual cycle. Blue arrows refer to the asexual cycle.

*U. necator*, an ectoparasitic ascomycete, is the causal agent of grapevine powdery mildew. This obligate biotrophic fungus can attack all photosynthetically active organs of the plant, such as buds, flowers, young berries, shoots, and stalks. This pathogen is mostly feared because it causes grapes to rot by acquiring nutrients through globose haustoria formed by the mycelium in epidermal cells. The disease associates to a white to grayish ashy powdery spot of a mealy consistency, which can later extend to cover an entire organ. The fungus overwinters as dark spherical cleistothecium (also defined as chasmothecium), the survival fruiting body usually present at the base of the vine barks or on fallen leaves [12]. The attack starts in spring on the young organs of the vine, when cleistothecia absorb water and crack, releasing mature ascospores (Figure 2). The infecting ascospores will produce a mycelium that, under suitable conditions, will grow on the epidermis of leaves (from 15 to 25 °C, minimum 2 mm of rainfall, 40% relative humidity, and 12 h of darkness) (Table 1). Basal leaves are infected first due to their proximity to the overwintered cleistothecia. Following infection, the mycelium can rapidly spread on green tissues, forming a pale white weft of hyphae.

When the mycelium reaches maturity (under favorable conditions, colonies begin sporulating after five days), asexual reproduction begins with formation of chained egg-shaped conidia on short erect conidiophores. Conidia are diffused by the wind (e.g., with a speed of at least 2.3 m s^−1^) and do not require leaf wetness as long as the moisture conditions of the atmosphere are relatively high [12,22,23]. With cooling weather, cleistothecia are produced mainly on wood following combination of the antheridium and oogonium of different mating types. Cleistothecia are partially buried in the mycelium weft on plant tissue and, when detached, retain myceloid appendages. Some cleistothecia may release ascospores in autumn, and, in some cases, the fungus can overwinter as mycelium in infected dormant buds, which will give rise in the next growing season to so-called flag-shoots, a source of secondary infection.

## 2. Analysis of the Literature

The literature search was performed on the Science Citation Index Expanded/Core Collection Database of Web of Science (WoS), and in Scopus. The SCIE is made available online through various platforms, and we queried the Thomson Reuters Web of Science (https://clarivate.com/products/web-of-science/, accessed on 24 November 2022). Scopus was accessed at https://www.scopus.com (accessed on 24 November 2022). The search statements (i.e., the queries that identify the information to be searched in the bibliographic database) were obtained by combining different terms with the Boolean operator “AND”. Specifically, we employed 19 terms divided into three item sets (G), G1 (*Vitis vinifera*, grape), G2 (downy mildew, powdery mildew, *Uncinula necator*, *Plasmopara viticola*, *Erysiphe necator*, *Oidium tuckeri*), and G3 (model, forecast, modelling, modeling, prediction, simulation, fungicide scheduling, forecasting, DSS, disease support system) for a total of 240 combinations (one term for G1, G2, and G3).

## 3. Results and Discussion

### 3.1. Quantitatve Descriptors of the Selected Documents

The database search provided a total of 466 documents. After removing duplicated entries and documents off topic (e.g., they did not focus on pathogen modelling, or referred to other pathogens), we obtained 199 documents. We also excluded non-English material, errata, and documents prior to 1981 or indexed as “conference paper”, for a final number of 110 documents (Table 2).

**Figure 3:**
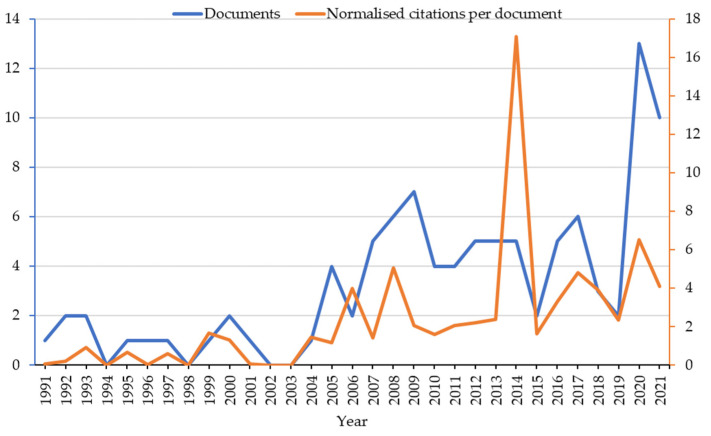
Evolution of main bibliographic indices throughout the years. The number of documents is in blue and the normalized average number of citations per document in orange.

The number of documents, from 53 different journals and books, has an annual growth rate above 7%. The literature showed a significant increase since 2000, with a second rise evident in the last few years (Figure 3). The recent growth was also implied by the average age of the documents (9.44 years). The normalized number of citations per document (i.e., the average number of citations divided by the number of citable years) also indicated an increasing interest in the sector, and its trend mirrored the number of publications.

**Figure 4:**
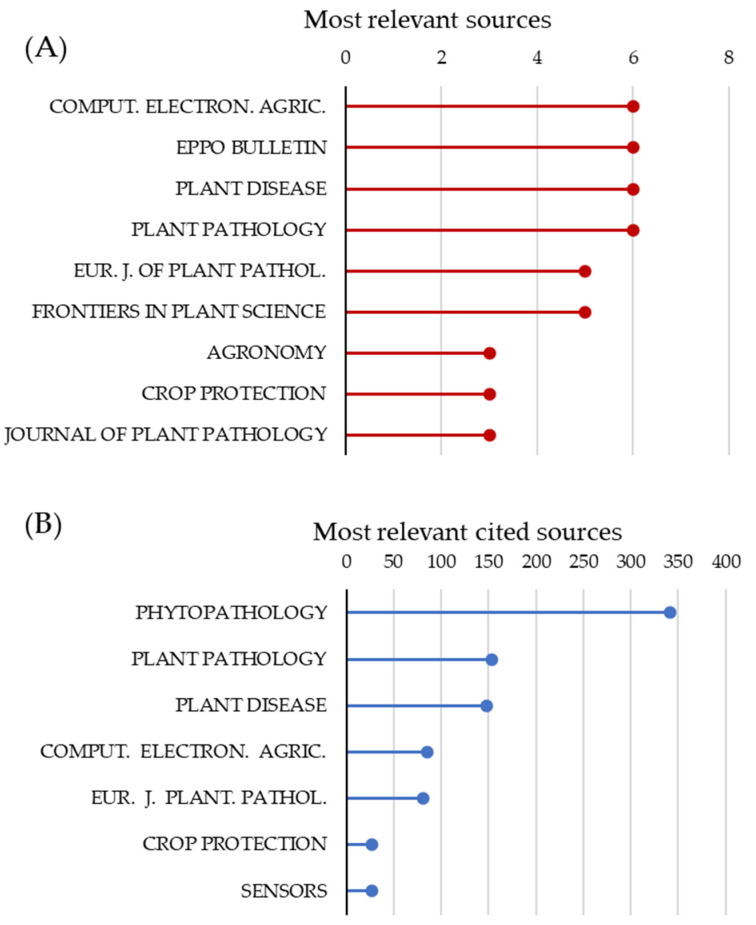
Most relevant sources (**A**) and cited sources (**B**) for the selected documents. To simplify the graphical representation, sources (respectively, most-cited sources) with less than four (resp., 25) documents are omitted.

Analysis of the most relevant sources well indicated the interdisciplinarity of the scientific area, with journals dealing with fundamental and applied plant pathology as well as development and application of electronics for solving problems in agriculture or plant science (Figure 4A). The most relevant cited sources were predominantly related to the field of plant pathology, underlying that the theoretical assumptions and the conceptual framework for modelling development mainly derived from information related to the study of plant diseases and pathogens (Figure 4B).

**Figure 5:**
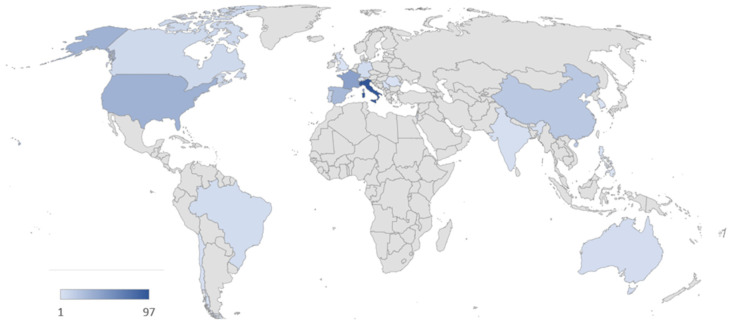
World map distribution of studies measured as the number of author appearances by country affiliation. The intensity of the blue color is proportionate to the number of papers, as indicated by the scale bar on the lower left corner.

The geographic distribution of the research, measured as the number of author appearances by country affiliation, indicated strong overlap between the countries involved in grapevine cultivation and vine production, with Italy and France being the most productive nations for the scientific field (Figure 5).

Collaboration among countries was measured in terms of co-authorship of the authors in the selected bibliographic documents and was graphically represented as a network (Supplementary Figure S1). As expected, the countries with a long history and larger production (i.e., Italy and France) were those with higher degrees of international collaboration. Moreover, the network illustrated that cultural and geographical affinities have clearly influenced collaborations.

Screening of the literature indicated that there are different kinds of data used for model development. For instance, among the climate data, the air temperature was widely used, probably because it is easy to measure and interpret in a biological context, being highly correlated with development of PM and DM in vineyards. Moreover, warning system models have grown to use different meteorological features, such as precipitation [24], humidity [25], leaf wetness with an hourly frequency [25,26,27], plant water stress [28], and others [10,29]. Field data and on-site measurements have also been employed to describe pathogen dynamics and modelling. They also explicitly consider the relationship between consecutive growing seasons. For instance, Rumbolz and Gubler [30] developed a model to determine primary infection of DM based on inoculation of shoots of grapevine in vitro and in fields in California, indicating that the incidence of infection on the outer bud scales strongly correlated to the flag shoot incidence in the subsequent season. The historical vineyard information for DM/PM disease modelling is also among the kind of data employed for model development. They often require accurate data on occurrence, incidence, and severity over long time series. However, this information is not frequently available while being critical in the model validation process [31]. Because of these issues, models based on historical information are more difficult to generalize and are expected to be useful particularly for a specific area. Regrettably, data gaps, inconsistent estimation techniques, and a lack of data quality assurance represent common limitations of databases and models [32]. Studies regarding disease recognition from visual data sources were limited. For instance, two studies employed RGB imagery to identify the beginning of DM symptoms and provide early warnings for their treatment [33,34]. Moreover, an approach based on algorithms for pattern recognition of grape leaf diseases (including DM) was described [35]. While some models specifically refer to the biological properties of the pathogen, others focus mainly on statistical assumptions. Among the latter, those based on generalized linear (mixed) models (GLM and GLMMs) and hypothesis testing are prevalent [9,36]. Among the models specifically developed for a pathogen, the most frequently considered were the mechanistic models described by Rossi, Caffi, and collaborators [10,37,38,39], as indicated by the number of publications and citations. Specifically, among our literature search, Rossi V. authored 22 documents, followed by Caffi T (15). Similarly, the most-cited authors were Rossi V. (26 citing documents) and Caffi T. (23).

Without having the aim and ambition to present all the models developed so far, in the following section, we describe the principles of selection of popular and/or representative models and some examples of their performance in terms of disease prediction and detection.

### 3.2. Main Models Present in the Scientific Literature

The ***Etat potentiel d’infection* (EPI)** is a widely employed, simple pathogen-specific model developed in France in 1983 for DM [40]. EPI predicts the potential impact of the pathogen and its ability to cause a primary infection, and it is based on two sets of equations [40,41]. The first part of the model focuses on the conditions that determine oospore maturation. It evaluates over a 10-day period the ‘potential energy’ (PE) of the pathogen using climatic data from 1 November to 31 March. The calculation is based on the difference between the air temperature and rainfall of the current growing seasons and historical reference values over a 30-year period. The second equation evaluates the infective capability of the pathogen (kinetic energy, KE). This parameter is calculated every day between 1 April and 31 August considering the monthly nocturnal average of relative humidity, the monthly temperature, the average diurnal relative humidity (between 10 am and 6 pm), and the average daily temperature. The sum of PE and KE provides the EPI value. Unlike other models, the EPI does not include precipitation as a factor in the kinetic equation, being mostly used to estimate risk at the end of winter [42]. Moreover, the dates to separate the calculations are fixed based on the empirical assumption that the overwintering stage of the pathogens ends each year very close to April. The EPI model is based on study of the conditions required for development of the disease over several years, assuming that, as more historical data are used, the averages will become closer to the average conditions required for development of the disease, thus allowing more accurate predictions [21]. Some authors use the stage of potential fungal growth to delay fungicide spray programs [43], although it is also possible to use it for this purpose when disease development predicted by the kinetic phase model is below average [21]. In a retrospective study conducted with historical meteorological data from 1970 to 1999 for the Portuguese Bairrada region, it was demonstrated that the EPI could halve the number of fungicide treatments [44]. Vercesi et al. [45] modified the model to extend the oospore maturation phase from March to June using meteorological data from Italy from 1989 to 1995. They observed that the model was effective for early stages of infection, although it missed low-risk alerts that later, through several cohorts, led to severe field infections. In other studies, application of phytosanitary products was reduced by up to an average 57%, with some cases where phytosanitary products were not applied [46]. A shared problem of mechanistic and empirical models is that they are difficult to generalize to environmental conditions different from those where they were developed. Specifically, the EPI model has reliably predicted epidemics of downy mildew in the Bordeaux region (France); however, it requires specific calibrations and modifications when used under different climatic conditions [42,47,48,49,50]. Egger et al. [51] proposed addition of leaf wetness to the EPI model as it could improve prediction for disease establishment and subsequent disease development [19]. Even though the EPI model has a good trade-off between complexity and performance, it requires collection of large amounts of historical meteorological data, and some authors have reported that it generates false negatives [6], while others have pointed out that the EPI model has a tendency to overestimate risk, especially of secondary infections [52,53]. Another model developed in the Bordeaux area (France) for DM is the ***Prévision de l’Optímum de Maturation* model (POM)** [41]. This model, like EPI, aims at predicting the ripening date of most oospores. The POM model is based on daily rainfall (from September to March), average monthly rainfall, and a standard daily rainfall threshold based on historical data, under the assumption that disease severity in spring is related to rainfall prior to oospore germination: the earlier the spore maturation is reached, the more severe the disease is expected to be in the growing season [11]. For development of this model, historical weather data were retrieved for the same area for at least the last 20 years (1965–1985). The ultimate purpose of the model is to calculate dynamic oospore maturation (DOM), which is the date when most of the oospores are mature [54]. Rouzet et al. [55] reported similar results to those obtained by Sung et al. [54], referring to temperatures in autumn and winter as key factors in suppressing oospore dormancy and concluding that less than 5–10 mm of rain over 3 weeks and low temperatures in early spring stopped their maturation. These authors also underlined the need to add modifications for daily use, and that oospore maturation after winter cannot fully account for disease epidemics. Studies in the Portuguese Douro wine region showed good results in some years, although, in other years, DM development in the field did not occur as expected by the model [44]. According to Caffi et al. [39], although both the POM and EPI models have been tested in a range of environmental conditions, in Italy, they have neither been sufficiently accurate nor robust to effectively manage fungicide application against DM. Growers traditionally follow the so-called **3–10 rule** developed by Baldacci in 1947 for the Lombardia region [56]. According to this model, the first treatment to control primary infection starts upon simultaneous occurrence of three conditions: (i) air temperature not less than 10 °C; (ii) shoots not shorter than 10 cm long; and (iii) a minimum of 10 mm rainfall within 24–48 h [29]. Subsequent treatments are calendar-based. The **Goidanich model** [57] can be considered an extension of the “3–10” rule that also considers weather data to guide treatments. The model is triggered by the 3–10 rule [58]. Once the risk alert is activated, the temperature and relative humidity are monitored daily for calculation of the daily percentage development of the infection incubation according to Goidanich’s tables [57] or later proposed mathematical models, such as the PLASMO [59]. When the accumulated value is equal to or greater than 100%, it is assumed that the zoospores have reached maturity and the first evidence of infection is expected to be visible on the leaves. After this event, the model is reset, and, when the environmental conditions described above are met again, the process will restart. The model is based on daily analysis of climatological features, but, recently, it has been revised to consider hourly progress of incubation and its confidence interval [6,10,60]. It has been shown that length of incubation period also depends on the host target organ and its age, as well as on ontogenetic resistance [61,62]. For instance, the length of the incubation period of the phytopathogen on berries is longer than on leaves [61]. Successive modifications from the original model proposed by Goidanich et al. [57] have improved the quality of the output data, but not much attention has been paid to input data, which are provided through weather stations. Regrettably, they usually present gaps in data submission and issues related to their calibration. This is a general concern for all models relying on meteorological conditions as data sources [63]. Finally, based on progressive oospore maturation data, Pedrazzini et al. [64] indicated that the first fungicide treatment should be applied when a rate of 80% was reached to reduce oospore development. To maintain a rate below 80% throughout the cycle, it is estimated that 8–11 fungicide applications will be needed [65]. The **demographic growth** (DG) model was developed by Chellemi and Marois [66] to study the evolution of cohorts of PM in Napa Valley, California. This model showed good results in Egypt [67]. The model focuses on the secondary infection cycle of *U. necator*, from germination until the end of sporulation. The DG is an algorithm that includes different factors, namely determination of the fungal germination rate (GR), penetration rate (PR), reduction in germination rate due to the presence of liquid water on the leaf surface (GRM), and reduction in the number of conidia produced per day due to the presence of liquid water on the host surface (SRM). All these features are functions of the average daily temperature [66,68]. Given an initial inoculum (density of spores), the model allows calculation of the daily germination, penetration, and colonization rates. As an example, the number of conidia produced per colony at day five is obtained considering the number of colonies present at that day and the temperature. The product of the conidia at that day (in the example, five) and the GR provide the number of conidia on the following day (day six). The simulation also includes estimation of the role of water on the host surface (SRM), and it is designed to follow fungal growth up to 35 days. A required element of the model is that it is necessary to establish an initial inoculum and the probability of deposition to calculate the GR [66]. The results from the California trial showed an optimum temperature of 22–26 °C for the growth rate of the pathogen populations, as well as a 49% reduction in population size because of liquid water on the leaf surface [66]. Although rain is necessary for ascospore release [69], other factors, such as temperature, would determine the number of ascospores released and successful subsequent infection [22,70,71]. Taking all this into account, simulations showed that causing an 80% reduction in ascospore concentration, and a 10-day delay in ascospore emergence due to early fungicide application (also in combination with other management methods), significantly contribute to reduction in future fungicide applications. This is important for medium- or late-maturing varieties as a delay in germination of ascospores could coincide with the veraison stage of the vine [67,72]. The **DMCAST** model was developed in Geneva (New York, NY, USA) for DM, and it is based on climatic data [73] using the same parameters as the POM model [10]. The probability of oospore maturation at a given date of the calendar year is calculated using the probability density function, where mean and standard deviation refer to the number of days required by oospores to mature, estimated based on the cumulative effect of rainfall from 21 September to 31 March. The latter is conditionally calculated considering the daily amount of rainfall, the monthly average of rainfall over a 30-year period, the minimum threshold for daily rainfall, and the number of rainy days in the current month. The model is triggered when the cumulative proportion of mature oospores reaches 3%, a threshold that was empirically determined considering the 1985–1992 interval. Then, the occurrence of primary infections is predicted using the daily temperature and rainfall, also considering the time to complete germination and sporangia survival. For the secondary infection cycle, the incubation period is determined according to hourly data of temperature, relative humidity, and leaf wetness, along with the hours of darkness during the growing season. The DMCAST model is considered more accurate in predicting infections from the sexual stage rather than secondary infection periods. In this respect, the model always correctly predicted primary infection for nine consecutive years in NY state [73] yet correctly predicted just above a fifth of the downy mildew infections using data from Italy, with an average of 42 days delay [47]. In a study developed by Kennelly et al. [74], they reported average success in accuracy of predictions made for DM during the years 2001, 2002, and 2003 using this model of 25%. A later study [75] reported that the model tended to overpredict spore viability by 25% throughout the day and concluded that this could be an explanation for failure to detect the disease in the field in earlier studies, while Pérez-Expósito et al. [6] reported that this model, as well as the EPI model, tended to underestimate the risk of infection, estimating that at least 30 years of climatological data would be needed to provide accurate predictions. As with previous models, this one also requires validation and calibration before the model can be used in different environments [47], although the model optimized by Rossi et al. [10] in Piacenza (Italy) with a fully mechanistic approach requires no calibration or correction and provides an accurate, detailed, and dynamic simulation of the sexual phase of *P. viticola* [38,47,76]. Finally, the mechanistic model developed by Rossi and collaborators [77] at the Università Cattolica del Sacro Cuore (**UCSC**; Piacenza, Italy) estimates the whole infection process (from oospore maturation until appearance of symptoms) by breaking it down into component pieces. It is based on air temperature, relative humidity, leaf wetness, and rainfall. Briefly, the model calculates the time when the first break of the oospore’s dormancy occurs (in reference to the 1st of January); the infection progress of the (primary or secondary) oospore cohort; and the time of symptoms onset. If the “3–10” rule can be considered the simplest empirical static model, most likely the UCSC is the most complex dynamic mechanistic approach. While it is expected to be flexible toward different agro-ecological zones, it requires advanced meteorological monitoring, which includes not only hourly updates on RH, air temperature, and rainfall but also timely and reliable data transmission, as well as measurement of leaf wetness [10]. Caffi et al. [39] reported in their study in Quebec that this model provided predictions of disease risk ranging from 6 days before to 3 days after infection. This model does not need calibration for uses in different environments as it has been successfully tested in different environments [42] and provides accurate, detailed, and dynamic simulation of the sexual stage. However, it has a higher degree of complexity than empirical models [6]. Aside from the mentioned DG and PLASMO, models have been developed also to specifically follow progression of the secondary infections of DM [78,79,80,81], with the common aim to guide fungicide treatment following a primary infection prediction (e.g., 3–10 rule; OiDiag-System, etc.) [82], the phenological state of the plants, or appearance of the first symptoms.

Overall, mechanistic pathogen-specific models divide the pathogen cycle as a series of interconnected biological states, whose parameters are initially set according to studies in controlled conditions. Especially for *P. viticola*, there is general agreement on identifying similar key stages for infection, which often concentrate on the weather requirements setting the time of the first seasonal development of oospores and spore survival during dispersal. In particular, the earliest models concentrated on the conditions that trigger germination of overwintering oospores, and then models also aimed to include environmental factors determining plant colonization. The conditions defining spore survival and dispersal (along with inoculation and incubation) are mainly seen as factors controlling secondary epidemics. This probably derives from the traditional view of a polycystic infection cycle starting from primary oospore and causing epidemics because of secondary infections deriving from exponential clonal propagation [83]. More recent pathogen-specific models refer to both the sexual and asexual phase of the pathogen under growing experimental evidence that epidemics involves different genotypes and that long-range migration of secondary sporangia is more limited than anticipated [84], although a case-by-case epidemic assessment is probably necessary to provide conclusive evidence for each agricultural setting and annual weather conditions [50].

**Statistical models** can be broadly defined as those that specify mathematical relations among features without an explicit attempt to consider the features of pathosystems. They are simple to apply and can predict systems without previous knowledge on biological or epidemiological mechanisms [9]. Mechanistic models differ from traditional statistical models because the structure of the mechanistic ones makes explicit assumptions about the biological mechanisms driving infection dynamics [85]. Regarding the most commonly used traditional statistic models, generalized linear models (GLM) or generalized linear mixed models (GLMM) have been employed to make comparisons between measures of PM severity and incidence over time in Israel [4], to monitor sporulation dispersal and disease incidence in Germany [19], and to determine the incidence and severity of DM on leaves and bunches in the Bordeaux region (France). They can achieve an accuracy higher than 75% in early prediction of severe disease and can reduce fungicide use by 50% [9]. Good prediction accuracy of disease incidence in leaves and bunches was obtained with regularized regression models (LASSO) [9]. Other authors have used correspondence analysis (CA) and principal components analysis (PCA) to study the maturation of DM oospores as a function of climatic features in the Bordeaux region, finding a correlation between rainfall in October and November and severity of infections the following summer [55]. Partial least-squares discriminant analysis (PLS-DA) for prediction of DM infections near Rome using climatic, agronomic, and phytopathological data showed an accuracy of 80% in the first year of analysis [86]. Calonnec et al. [87] used a spatio-temporal logistic model in Bordeaux to analyze variation in disease intensity with time and distance from the source of the initial inoculum by exploiting the sigmoidal growth trend typical of pathogens due to constraints such as space or resources, while Patil and Thorat [88] developed a hidden Markov model (HMM) supported by an IoT system on farms in India to identify and anticipate DM and PM infection, achieving a 91% accuracy. Thanks to the rise in computational power and development of specific libraries in different programming languages, several authors have tried to tackle plant disease detection through **artificial intelligence (AI) models**, a broad group of tools or algorithms that select an output among possible alternative options through acquisition of knowledge and manipulation of information. According to the comparison by Baker et al. [89], AI models differ in various aspects from mechanistic models. The latter seek to establish a mechanical relationship between inputs and outputs, do not allow for an agile way to accurately incorporate information from multiple spatial and temporal scales, are able to handle small datasets, and, once validated, can be used as a predictive tool where experiments are difficult or costly to perform. AI models seek to establish mathematical relationships and correlations between input and output, can address problems with multiple spatial and temporal scales, but require large datasets to be trained and can only make predictions related to patterns within the supplied data (unless they are provided with new data entries). Among the most widely used AI models for classification of different disease levels of DM and PM in the field, there are supervised learning classification algorithms, such as random forest (RF), gradient boosting (GB), support vector machines (SVM), convolutional neural networks (CNN), probabilistic neural network (PNN), or deep learning algorithms. The main models found in the vineyard literature are based on diagnosis based on RGB images, multispectral images, or radio frequency signals. In a study by Sandika et al. [90] in India, it was observed that the model that best classified the disease indices of both downy and powdery mildew was RF, with 82.9% accuracy over PNN, backpropagation neural network (BPNN), and SVM models. These results were supported by the study of Knauer et al. [91], in which they obtained 87% accuracy in detecting powdery mildew in Chardonnay grapevines from South Australia. Shruthi et al. [92] used a wide variety of plant species in their study and observed average accuracies for grapevine diseases of 88.9% for SVM and 96.3% for CNNs. Wang et al. [93] showed accuracies above 90% using SVM, PNN, and neural networks together with other techniques in vine diseases. However, acquiring disease data through image analysis is not the only way in which it is possible to predict diseases. In their work in Bordeaux, Chen et al. [9] collected data visually, recording incidence and severity rates on a weekly basis and relating these observations to the environmental data to which the vines were subjected. They concluded that both RF and GB models showed different accuracy in classifying the incidence of the disease depending on the features introduced into the model. For instance, the GB model obtained area under the curve (or AUC, ranging from 0 to 1) values of 0.86 using both date and meteorological data. However, using only meteorological data, the AUC value dropped to 0.70 in the case of the RF model. Finally, there is a model derived from the PNN that combines Bayesian statistics and neural networks to generate event probabilities given certain conditions (Bayesian neural network). Although initially this model could be applied to any pathogen, the equations on which the work developed by Lu et al. [94] aims to calculate primary and secondary infection rates focus on the dynamics of growth and dispersal of the PM. The model proposed by these authors is mainly based on calculation of the progression of the different biological stages of the fungus [36,78] as a function of temperature and on the assumption that the probability of occurrence of some of them depends on the values of the previous ones. Thus, a network of interdependence of the features is created so that it is possible to train a model based on the detected trends and finally obtain a conditional probability of the last feature present in the network, which totally depends on all the previous ones (in this case, the disease incidence (DI) obtaining relatively low mean absolute error (MAE) values). Other works have been published using Bayesian networks, but they have not yet been applied in grapevine [95,96,97].

## 4. Conclusions and Perspectives

The first conclusions that can be drawn from the literature screening are that the years from the 1980s until the early 2000s were characterized by mechanistic and statistical models, and AI models started to be more represented after 2010. Moreover, while the earliest models were pathogen-specific, the literature was later populated also by more generalist and easier-to-apply generalist models, which do not involve initial empirical development. Another implication is that many authors mention in their manuscripts the need for model validation in other regions of the world as the models have often been trained and tested in one locality. While it is widely acknowledged that model accuracy may decrease in other agro-climatic environments, the increasing number of models and related publications indicated that there is more interest in developing alternative solutions rather than refining existing ones, although this conclusion may be biased by our focus on academic literature. Considering the rising economic importance of grape mildews, it may be possible that diffusion of non-empirical approaches derives from the possibility of translating knowledge from herbaceous plants (such as tomato, cucumber, or rice) and other stresses (e.g., water stress, bacteria, viruses). These new studies further extend the type of algorithms used to include models, such as K-nearest neighbors (KNN), naïve Bayes (NB), or classification and regression trees (CART). Considering the good accuracy metrics of the AI models studied for grapevine and other plant species, it seems that this trend will play an important role in the short term. However, the available literature does not allow to firmly conclude whether research should point more towards individual pathogen-centered solutions or the “one-model-fits-all” approach because they are both popular in the recent literature.

This systematic analysis also allowed us to justify major challenges and perspectives for grapevine farming. There is room to improve access for scientists, developers, and growers regarding consistent weather data with appropriate spatial and temporal scale. Considering the biology of the pathogens and plant response, our literature study confirmed that this information has been and will be at the core for development and validation of local, regional, or national models. Temperature and rainfall are the most used features, and it is, therefore, worthwhile to recall that two main effects of climate change are increased temperatures and variability in rainfall seasonality and intensity [98]. A first concern is that computational predictions related to northern Italy already indicated that the number of fungicide treatments to control DM will increase, with a possible boost in primary infections up to June, implying the necessity of increased precision and capability of forecast models [99]. In addition, it has been considered in different contexts that disease prediction will greatly benefit from a large increase in the scale resolution of the weather data in space (e.g., from in-field monitors) and time (up to the minute) [100].

Although significant progress has been achieved, the need for integrated multi-model ensembles for disease predictions has not been fully met, especially to provide robust decision-making and to be easily accessible and hopefully transferable to various agricultural settings. Integrated pathogen prediction systems should also be aligned to climate modelling and observational studies, but, so far, they have almost inexplicably insufficiently considered plant status and response to pathogens despite the archetypal knowledge that disease development is ruled by the so-called triangular interaction between host, pathogen, and environment. Under this perspective, an integrated predictive solution should refer also to field studies spanning from molecular diagnostic screening to ‘multi-omic’ approaches to disease [101], from quantification of the initial hallmarks of disease to identification of a risk profile for grapevine genotypes [102,103]. Possible additional factors for model development should also be able to identify elements that induce crop damage (and not only pathogen epidemics) and ascertain the directionality of relevant changes in pathogen population dynamics, also following treatments. Recent developments in computer science and hardware engineering provide solid hope of being able to completely understand grapevine risk during the whole growing season. This could be achieved exploiting analysis of data and information collected and transmitted through advanced digital tools [104,105]. Additionally, it would be beneficial to integrate data from portable molecular diagnostic tools to independently validate prediction models and, therefore, reveal insights necessary to fine-tune timely intervention in vineyards (i.e., spore monitoring, pathogen detection, effects of treatments) [106]. Finally, it is also worthy of consideration that international cooperation in the sector should be improved. While collaboration between nations that have a long history of developing predictive systems (Italy, France, and USA) is noteworthy, studies with emerging wine-countries wishing to develop capacities are more limited.

## Figures and Tables

**Table 1:** A summary of the main factors that favor growth and dispersion of *P. viticola* and *U. necator* diseases on grapevine and range of time to infection.

Disease	Rainfall (mm)	Temperature (°C)	Relative Humidity (%)	Windspeed (m s^−1^)	Time to Infection ^1^ (Days)
Downy mildew	6–10	6–26	>90%	>9.0	7–18
Powdery mildew	2–10	15–25	>40%	>2.3	5–7

^1^ From opening of resistance structures to completion of primary infection.

**Table 2:** Main descriptors of the retrieved documents employed for this review.

Descriptor	
Documents	110
Sources	53
Year of the first document	1991
Annual Growth Rate (%)	7.35
Document Average Age (years)	9.44
Average citations per document	25.1
Authors	378
International co-authorships (%)	18.18

## Data Availability

Data of the literature search will be made available from L.V.-C. upon request.

## Supplementary Materials

The following supporting information can be downloaded at: https://www.mdpi.com/article/10.3390/microorganisms11010073/s1, Figure S1: Collaboration network among countries. The network was generated using the Fruchterman–Reingold layout algorithm based on the co-authorship in the selected bibliography. The size of each vertex is proportional to its degree of connection. The network was obtained with the igraph package in R 4.2.
