# Supplementary figures and images for: Current Trends and Perspectives on Predictive Models for Mildew Diseases in Vineyards
Source: Microorganisms. 2022 Dec 27;11(1):73. doi: 10.3390/microorganisms11010073 (PMC9866057; doi:10.3390/microorganisms11010073)

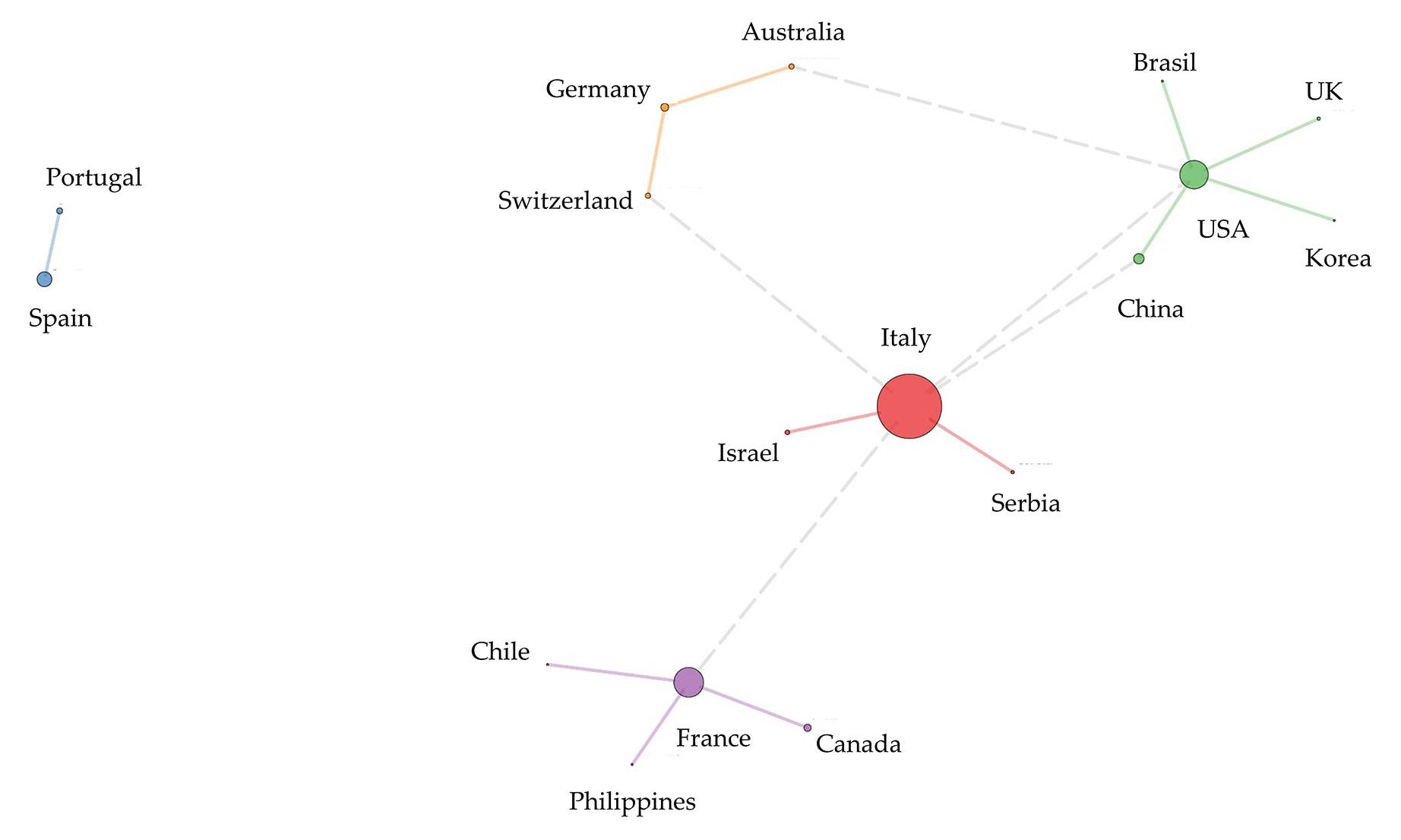

Supplement: Supplementary file 1 [file microorganisms-11-00073-s001.zip › Figure S1.jpg]
